# Genome Sequence of *Lactobacillus* *sunkii* Strain CG_D

**DOI:** 10.1128/genomeA.01487-16

**Published:** 2017-01-12

**Authors:** Christina Gabris, Anja Poehlein, Frank R. Bengelsdorf, Rolf Daniel, Peter Dürre

**Affiliations:** a Institut für Mikrobiologie und Biotechnologie, Universität Ulm, Ulm, Germany; b Genomic and Applied Microbiology & Göttingen Genomics Laboratory, Georg-August University Göttingen, Göttingen, Germany

## Abstract

*Lactobacillus sunkii* CG_D is a rod-shaped, Gram-positive, and heterofermentative lactic acid bacterium. The draft genome of *L. sunkii* strain CG_D comprises 2,794,637 bp with an average G+C content of 42.03%. The genome harbors 2,662 predicted protein-encoding, and 71 RNA genes.

## GENOME ANNOUNCEMENT

*Lactobacillus sunkii* was isolated in 2009 from sunki, which is a traditional Japanese pickle (1). *L. sunkii* is a Gram-positive, rod-shaped, and heterofermentative lactic acid bacterium. Species of *Lactobacillus* are not only used in the food industry for pickling processes or yogurt production, but they are also known as common silage inoculants to ensure stability of the silage (2). Additionally, 16S rRNA gene sequences of the genus *Lactobacillus* were found in microbial communities of biogas reactors (3, 4). Here, we present the draft genome sequence of the *L. sunkii* CG_D strain, which was isolated from biogas reactor content and initially designated *L. sunkii* CG01 (5). The biogas reactor was fed with maize silage and cattle and poultry dry manure and is located in Troisdorf, Germany (6).

Genomic DNA was isolated from *L. sunkii* CG_D cultured in RCM liquid medium (Oxoid Ltd., Basingstoke, Hampshire, United Kingdom) at 37°C under an N_2_ and CO_2_ (80% + 20%) atmosphere. For genome sequencing, we used a combined approach using both the Titanium chemistry of the 454 GS-FLX pyrosequencing system (Roche Life Sciences) and the Genome Analyzer IIx (2- × 112-bp paired-end sequencing; Illumina). Sequencing libraries were generated from the extracted genomic DNA according to the instructions of the manufacturer (Illumina, San Diego, CA, USA; Roche Life Science). Sequencing resulted in 10,685,282 paired-end reads (112 bp) that were trimmed using Trimmomatic 0.32 (7) and 101,422 reads generated by 454 pyrosequencing. Mira v 3.4 (8) was used for the hybrid *de novo* assembly, which resulted in 40 contigs with an average coverage of 376-fold. The genome of *L. sunkii* CG_D comprises presumably one circular chromosome of 2,794,637 bp with an average G+C content of 42.03%. For automatic gene prediction, the software tool Prodigal (9) was used. Genes coding for rRNA and tRNA were identified using RNAmmer (10) and tRNAscan (11), respectively. The Integrated Microbial Genomes-Expert Review (IMG-ER) system (12) was applied for automatic annotation followed by manual curation using the Swiss-Prot, TrEMBL, and InterPro databases (13).

The genome was shown to encode at least 49 predicted RNAs, including three rRNAs and 46 tRNAs. A total of 2,711 identified genes yielded a coding capacity of 2,442,225 bp (coding percentage, 97.39%). Among the 2,662 protein-encoding genes, 1,930 (72.50%) were found as putative proteins with function prediction and 732 (27.50%) were assigned as hypothetical proteins. We performed *in silico* pairwise ANI average nucleotide identity (ANI) (14) with the genome sequence of the type strain *Lactobacillus sunkii* DSM 19904^T^ (version 4.540 deposited on IMG/ER) (12). A seqeucne similarity of 96.28% showed that the isolate CG_D belongs to the species *L. sunkii* (14). Genome analysis showed that two genes encoding a xylulose-5-phosphate/fructose-6-phosphate phosphoketolase, the key enzyme for heterofermentative lactic acid fermentation, are present. We also identified genes encoding phosphotransacetylase and acetate kinase as well as several genes encoding alcohol dehydrogenase and l-lactate dehydrogenase. Moreover, growth experiments showed acetic acid, lactic acid, and ethanol production (5).

### Accession number(s).

This whole-genome shotgun project has been deposited at DDBJ/ENA/GenBank under the accession no. MIQE00000000. The version described in this paper is version MIQE01000000.
